# A study protocol for a quasi-experimental community trial evaluating the integration of indigenous healing practices and a harm reduction approach with principles of seeking safety in an indigenous residential treatment program in Northern Ontario

**DOI:** 10.1186/s12954-021-00483-7

**Published:** 2021-03-17

**Authors:** T. N. Marsh, C. Eshakakogan, J. K. Eibl, M. Spence, K. A. Morin, G. J. Gauthier, D. C. Marsh

**Affiliations:** 1 Northern Ontario School of Medicine, Sudbury, Canada; 2 North Shore Tribal Council, Cutler, Canada; 3 Health Sciences North Research Institute, Sudbury, Canada; 4 ICES North, Sudbury, Canada; 5 Canadian Addiction Treatment Centres, Markham, Canada

## Abstract

**Background:**

Indigenous communities in Canada face significant challenges with intergenerational trauma, which manifests in substance use disorders. There is consensus that connecting treatment approaches to culture, land, community, and spiritual practices is a pathway to healing trauma and substance use disorders for Indigenous peoples. Indigenous residential addiction treatment programs have been established as the primary intervention to provide healing for Indigenous peoples with substance use disorders and intergenerational trauma. However, there is limited evidence demonstrating the effectiveness of these programs. In collaboration with the Benbowopka Treatment Centre, this paper describes a study protocol which aims to evaluate the effectiveness of blending Indigenous Healing Practices and Seeking Safety for the treatment of Indigenous patients with intergenerational trauma and substance use disorders.

**Methods:**

We will conduct a pre/post Quasi Experimental Community trial, to compare historical treatment outcomes for patients following the implementation of Indigenous Healing and Seeking Safety. We will conduct quantitative and qualitative analyses to understand the differences before and after the intervention is implemented. The pre- Indigenous Healing and Seeking Safety intervention study window will span from 2013 to 2016; *n* = 343, and the post-Indigenous Healing and Seeking Safety intervention study window from 2018 to 2020; *n* > 300. All participants will be enrolled in the Benbowopka residential treatment for the first time during the study periods. All data will be anonymized at the time of data entry. Propensity matching will be undertaken for patient characteristics, including sex/gender, age, and substance use type.

**Results and conclusions:**

The study findings could be used to inform intergenerational trauma and substance use disorders residential treatment programming for Indigenous communities across Canada. Our work will contribute to the field of community-based intergenerational trauma and substance use disorders programming by addressing objectives that consider: (a) the patient perspective, (b) the program perspective, and (c) the community perspective. The study findings may validate an innovative approach for evaluating the effectiveness of residential addiction treatment and particularly the effective and appropriate care for Indigenous patients with intergenerational trauma and substance use disorders.

## Background

Approximately 474 000 Indigenous people live in 617 First Nations communities across Canada; 125 of those communities are located in Ontario, primarily in rural and remote areas [1]. The remoteness of these communities, as well as rural health challenges, coupled with the lack of traditional and culturally sensitive treatment models pose huge challenges for the treatment of intergenerational trauma (IGT) [2–5]. These factors have strongly contributed to the multigenerational grief and loss associated with IGT [5–8].

Today, many Indigenous communities affirm that their healing and wellness is embedded in reclaiming cultural and Spiritual interventions. These include traditional teachings, presence of Elders, sweat lodges, prayers, fasting, and sacred song and dance [9–12]. Indigenous treatment programs in urban and regional areas integrate these cultural practices into programs [13]. In Canada, for example, the 56 National Native Alcohol and Drug Abuse Programs (NNADAP) and nine Youth Solvent Addiction Program treatment centers support and encourage Indigenous traditional healing practices and culture as an integral part of health, wellness, and healing [14]. These programs are overseen by First Nations communities and support a network of residential treatment and community prevention programs practicing Indigenous traditional healing practices, Spirituality and creation stories [15–17].

Between 2004 and 2009, the number of Indigenous patients seeking treatment for substance use disorders (SUD) in Ontario increased threefold [18]. The Chiefs of Ontario have identified opioid use disorder as a health priority as the number of people seeking treatment continues to climb (Chiefs of Ontario, 2009). Many Elders, traditional healers, and Indigenous scholars agree that connecting to culture, land, community, and spiritual practices is a pathway to healing IGT and substance use disorders (SUD) for Indigenous peoples [19–21]. The skills required to heal from IGT and subsequent recovery of identity can be enhanced by cultural practices, Indigenous Healing (IH), the presence of Elders, and traditional ceremony [5, 20, 20, 22, 23].

Outpatient and residential treatment of SUD within Indigenous communities presents many challenges and barriers [2, 24]. Many communities continue to report the need for increased access to evidence-based treatment options [25, 26]. While it appears as though some success has been achieved by treatment models [24, 27, 28]; there is a lack of access and a need for evidence-based treatments [24, 27, 28]. Many communities report a lack of services, specifically Indigenous treatment options and culturally sensitive approaches, as well as a lack of cultural awareness from health care professionals and rehabilitation services [25]. Moreover, there is currently limited knowledge in regard to how well the existing services and treatment centers in northern Ontario are addressing the needs of young people with SUD.

In Canada, there are 49 federally funded adult and youth NNADAP and nine YSAP treatment centres. The majority of NNADAP centres were founded on Western approaches to treatment in the 1980s, and later incorporated Indigenous understandings of healing and personal growth. Therefore, to date the majority of addiction treatment for Indigenous populations in Canada is residential treatment [14]. An addictions service needs assessment study conducted in Ontario First Nations communities—the majority of which were located in northern Ontario—reported that the addiction-related education, promotion and prevention programs in their communities mainly targeted adults [29, 30]. These communities also report the effects of both IGT and SUD and the need for culturally based and culturally safe services, particularly those that are informed by institutional IGT experienced by Canada’s First Nations, Métis and Inuit peoples [29, 31].

De Andrade et al. 2019 conducted a systematic review of all studies on the effectiveness of residential treatment published between January 2013 and December 2018 [32]. They identified that despite the growing need for effective residential SUD treatment internationally, there was a lack of consensus on best practice treatment guidelines. In line with previous reviews, this review is of the most recent studies in the field (2013–2018) and provided some evidence that residential treatment may be effective in reducing substance use and improving mental health [32]. Furthermore, the authors found evidence that treatment may have a positive effect on social and offending outcomes. This research also identified an urgent need to conduct more research in this field that could address significant methodological flaws, and affirm that these flaws could be partially overcome by the use of data linkage practices to monitor outcomes [32]. Of note, there was an absence of evidence evaluating the impact of residential addiction treatment on broader health system utilization.

In a recent paper by Munro et al. 2017, the authors identified that residential rehabilitation is not just about length of time and client characteristics in treatment. Culture, ceremony, activities and connections were also integral to the therapeutic healing process and these activities were found to enhance positive change in clients over time [33]. Furthermore, this study reported that the cultural elements offered hope and healing for clients in a remote Indigenous residential addiction treatment program [33].

Other researchers and authors affirm that effective, culturally safe residential rehabilitation services for Indigenous peoples are crucial to enhance recovery and success in treatment outcomes [34]. However, research, evaluation and science inquiry requires a sound theory in combination with rigorous testing and validation of tools. There is a lack of this required evidence in the Indigenous addiction treatment literature. Such studies in Indigenous populations have been criticized due to a reliance on small sample sizes [16, 35].

Recent work from Dr. Teresa Marsh has demonstrated that Indigenous and Western approaches for the treatment of Indigenous patients with a history of IGT and SUD could be effectively combined with positive results using the Indigenous Healing and Seeking Safety (IHSS) approach [24, 36]. The Western Model, Seeking Safety (SS), is a psycho-educational counseling program that targets the unique problems resulting from SUD and IGT [37]. SS incorporates the inclusion of the mind, body, spirit, and self-awareness during treatment, as well as connection to community through an emphasis on the utilization of community resources. The intervention aims to increase the coping skills of participants and reduce the chance of relapse by emphasizing values such as respect, care, integration, and healing of self [38]. Seeking Safety has been translated into 14 languages and evaluated in over 40 published studies [39]. The treatment has shown positive results and is the only model for PTSD/SUD thus far to impact both PTSD and SUD [39]. Also, Seeking Safety has been implemented with Indigenous peoples in Canada and showed positive results [40]. Importantly, the perspective of SS is convergent with Indigenous traditional healing methods. Due to the content and delivery method of SS, the program complements traditional teachings such as holism, relational connection, spirituality, cultural presence, honesty, and respect [15, 16, 24, 41].

Benbowopka Treatment Centre started in 1991 as a 12-Step Alcoholics Anonymous model of intervention to address alcohol addiction that was prominent in many First Nations communities during that time. However, the onset of prescription drug misuse in First Nations communities shifted the need for addictions treatment towards harm reduction models of service. This has prompted Benbowopka Treatment Centre to go through a significant transformation over the last 36 months in regard to ensuring its services are better able to address the SUD needs of First Nations communities.

The abstinence model of service that was being delivered at Benbowopka Treatment Centre was excluding many individuals who were seeking residential addictions treatment for problems related to prescription opioids and other drugs. Individuals who were on prescribed medication, including opioid agonist treatment, by their physician to address SUD or mental health challenges were not eligible for admission to Benbowopka Treatment Centre because of its abstinence-based model of service. In 2014, Mamaweswen North Shore Tribal Council directed Benbowopka Treatment Centre to move forward with a plan for the realignment of its services to a harm reduction model of service that would follow the best practice strategies recommended for addictions treatment. Thus, the implementation of the Indigenous Healing and Seeking Safety (IHSS) approach at Benbowopka for Indigenous patients with a history of IGT and SUD was initiated in 2016.

The Mamaweswen (North Shore Tribal Council) and the Benbowopka Treatment Centre represent seven member communities (Batchewana, Garden River, Thessalon, Sagamok, Mississauga, Atikameksheng, and Serpent River) on the North Shore of Lake Huron. Benbowopka treats approximately 90 patients per year in a residential treatment program. The program has high-quality retrospective data based on standardized assessment screening tools routinely used by the Drug and Alcohol Treatment Information System and the National Native Alcohol and Drug Abuse Program.

This study will aim to evaluate the effectiveness of the validated treatment model (IHSS) for the treatment of Indigenous patients with a history of IGT and SUD in a four-week residential treatment centre. Study findings will be used to inform trauma and SUD residential treatment programming and implementation for Indigenous communities across Canada. Hopefully this work will contribute to the field of community-based trauma and addiction programming by addressing objectives that consider:

1. *The patient perspective* does patient satisfaction improve when IHSS methodology for treatment of trauma and SUD replaces an abstinence-focused approach?
2. *The program perspective* do program completion rates improve following the implementation of IHSS intervention?
3. *The community perspective* does the revised program serve a broader range of community members requiring treatment for SUD and IGT? Does the impact of residential addiction treatment on reducing broader health care utilization (through reduced substance use related emergency room presentations and hospital admissions in the year following treatment completion) increase following IHSS implementation?

## Methods

### Aim

The aim of this study will be to determine whether patients who are treated with the IHSS model will have improved treatment satisfaction (increase of 10% over pre-intervention rates), improved treatment completion (increase of 10% over pre-intervention rates), and a greater reduction in health system usage (substance use related presentations to emergency room and hospital admissions) at one-year follow-up compared to those treated within the abstinence-based model (decrease of 10% over pre-intervention rate).

### Design

We will conduct a pre/post Quasi Experimental Community trial in northern Ontario, Canada, comparing historical treatment outcomes for past Benbowopka patients to outcomes following the implementation of IHSS treatment for Indigenous patients with a history of IGT and SUD. We will conduct a quantitative and qualitative analysis to understand the differences before and after the intervention is implemented. The pre-IHSS intervention study window will span over 36 months (April 1, 2013–March 31, 2016; *n* = 343), and the post-IHSS intervention study window will span the 27 months post-IHSS implementation (January 1, 2018–March 31, 2020; *n* > 300). We conducted an a-priori sample size calculation for our study with an anticipated effect size of 0.5, and probability level of 0.05. To have appropriate statistical power of > 80%, we would need 65 patients in each group. With the anticipated sample sizes of 300 pre and post-implementation, we have enough statistical power to confidently evaluate our outcomes.

All patient data will be anonymized at the time of data entry. Propensity score matching will be undertaken to evaluate the association between the IHSS treatment and long-term outcomes. The National Native Alcohol and Drug Abuse Program (NNADAP) tool will be used to analyze the program objective; that is, whether completion rates improve following the implementation of the IHSS intervention. Additionally, a Client Quality Assurance Survey will be utilized to determine patient satisfaction.

### Setting of the study

Benbowopka treats approximately 90 patients per year in a residential addiction treatment program in Blind River Northern Ontario. The study periods will include pre-intervention (April 1, 2013–March 31, 2016) and the post-IHSS intervention study window spanning the 27 months post-IHSS implementation (January 1, 2018–March 31, 2021; *n* > 300). Time periods will be chosen to allow analysis by both calendar year and fiscal year. On every 28-day treatment cycle, participants will be recruited and informed by the staff at Benbowopka about the study and, if interested, a consent will be obtained. The data collection will include data from patients initiating treatment at Benbowopka during the study window. Patient follow-up will occur in the year following treatment completion and/or discontinuation. Anonymized patient records will be linked via Ontario Health Insurance Plan (OHIP) number to the administrative health data at the ICES. ICES, formally known as the Institute for Clinical Evaluative Sciences, is an independent research institute that collects and analyzes data on publicly funded health services for research. The data will be queried in the one year following completion or discontinuation of IHSS at Benbowopka to ascertain the impact of substance use on health outcomes including hospitalization, emergency department visits and opioid-related overdose. For patients with multiple admissions, the date of first admission will serve as the index date for health outcomes evaluation.

### Study population

We will only include data from first-time patients initiating treatment at Benbowopka, meaning that there will be no previous history of treatment at this center. It is common for patients to cycle between treatment and relapse. Studies have shown that multiple treatment attempts are associated with a higher likelihood of positive outcome [32]. Therefore, we will only include first-time patients to eliminate bias associated with cases involving multiple treatment attempts.

Benbowopka’s residential treatment program collects individual-level data based on standardized assessment screening tools routinely used by the Drug and Alcohol Treatment Information System and the National Native Alcohol and Drug Abuse Program. The cohort for this study will be created by using anonymized patient records from the Ontario Health Insurance Plan (OHIP) number. We will link the OHIP number to the administrative health data at ICES in order to be able to evaluate broad health outcomes for patients after they completed the treatment program. ICES routinely links partner data to its core health systems data holdings. We will then query data in the one year following completion or discontinuation of IHSS at Benbowopka to ascertain *Impact of Substance Use on Hospital Costs* as defined by the Canadian Center on Substance Abuse [42]. ICES linkage will enable virtual follow-up of Benbowopka patients for health systems usage. A detailed timeline can be found in the appendix.

### Data governance and ethical considerations

We will respect the Tri-Council Policy Statement, Chapter 9, which highlights the importance of engaging with First Nations throughout all phases of the research process. In addition, we will honour Indigenous knowledge by engaging with Elders and the Mamaweswen Council. Anonymized data collected at Benbowopka will be linked in accordance with data governance protocols and the data-linkage process will be established at ICES in consultation with the Chiefs of Ontario. All parties involved in the study will respect Indigenous principles of OCAP [43].

### Ethics

This research will be conducted in keeping with the Canadian Institutes of Health Research (2011) *Guidelines for Research Involving Aboriginal People* and the *Tri-Council Policy Statement for Ethical Conduct for Research Involving Humans* [44]. The study received approval from Laurentian University’s Ethics Board in May 2017.

### Training

Wellness, training, and capacity building will be the cornerstones of this research. Clinicians at Benbowopka were trained in the IHSS methodology (beginning January 2016) and IHSS is now routinely delivered to patients.

The five days of training on the IHSS program incorporates all of the elements of an integrative Two-Eyed Seeing and blended approach [45]. The training consists of didactic, experiential, and small-group learning. During the training the clinicians engage in IHSS sharing circle sessions such as Post Traumatic Stress Disorder (PTSD), taking back your power, setting boundaries in relationships, dealing with anger, and taking good care of yourself [46]. The design and delivery of this training is consistent with previous experience training IHSS facilitators by this research team [36, 47]. During these sharing circles the Elder offers a Grandfather teaching, Indigenous spiritual and traditional sayings, smudging, and/or prayers. The teachings of the Seven Grandfathers, also known as either the Seven Teachings or Seven Grandfathers, are a set of teachings on human conduct toward others. They include the concepts of wisdom, love, honesty, respect, bravery, humility, and truth [48]. The day starts with didactical presentations until lunchtime, and in the afternoon the clinicians engage in an actual IHSS sharing circle facilitated by the first researcher and an Elder. The clinicians have opportunities to ask questions, and through this in-depth discourse evolve. Day three and four focus on substance use disorders and exploration of information in the Seeking Safety manual. In-depth discussions help clinicians understand how the Seeking Safety sessions are offered as well as the integration of traditional healing practices. The last day of training focuses on vicarious traumatization, practice sessions, planning and implementation.

The investigators will work with the Benbowopka team and their digitized data collection health record system. The enhancement of the digital health record could enable efficient data capture and evaluation for quality improvement, research, and reporting purposes. Benbowopka staff, patients, and the Mamawesen communities will benefit from this training and technology enhancement going forward. Importantly, the reciprocal training and skills developed by the community and research team will strongly position collaboration for future community-based research studies and will materially increase the capacity for high-quality Indigenous wellness research in northern Ontario.

Community-based trainees include three clinical staff, a graduate student, and a research assistant. The academic research team will benefit from enhancing its understanding of Indigenous Healing, unique community perspectives, and Indigenous ways of knowing.

### Data collection

The historical program data will be collected from Benbowopka’s records from January 2013 to March 2016 (end of the 2015 fiscal year). The data will be divided into fiscal year and intake period at the request of Benbowopka. Historical demographic data for each intake period will be found in the “Client Information Summary” folders in Benbowopka’s archive room.

We will use the Drug Use Screening Inventory (DUSI) to collect data on patients during the program. The DUSI and a revised version (DUSI-R) were developed by the Center for Addiction and Mental Health (CAMH) to identify consequences of alcohol and drug involvement [49]. The DUSI is a 149-item multidimensional instrument that quantifies not only involvement with drugs and alcohol, but also associated problems in the areas of mental health and psychosocial domains. The DUSI-R consists of 159 items. The following variables will be collected: name, health card number, date of birth, postal code, status card number, gender, date of admission to the program, date of discharge, program completion (Y/N), Indigenous (Y/N), status First Nation (Y/N), on reserve (Y/N), primary substance, and secondary substance(s). Drug classes from primary and secondary substances will be further divided into a sub-substance category. Any pertinent notes will also be included in the data.

The original data will include drug classes used by Benbowopka (i.e. alcohol, narcotics, prescription drugs, solvents/inhalants, and other). The revised data will be organized by the drug class preference of the research team (i.e. alcohol, cannabis, stimulants, opioids, depressants, hallucinogens, inhalants/solvents). Some drug names will be changed to ensure cohesion (e.g. brand to generic names, acid to LSD, etc.).

### Administrative data variables

The administrative data will be collected by submitting a formal requisition to ICES. All diagnostic information from physician visits will be determined using billing data from OHIP. Emergency department visits will be identified using the National Ambulatory Care Reporting System (NACRS). Hospital admissions will be identified using the Discharge Abstract Database (DAD). We will obtain patients’ location of residence and demographic information, including all-cause mortality from the Ontario Registered Persons Database (RPDB), which contains unique data for each resident who has ever received insured health.

## Choice of outcome tools: quantitative and qualitative

### Data analysis

We will evaluate the results of the IHSS intervention against three distinct primary outcomes. The lens will be applied to these outcomes considered:

1. *The patient perspective* we will use Client Quality Assurance Survey tool to assess the **appropriateness** and **satisfaction** of the intervention
2. *The program perspective* we will use program completion as a primary outcome to assess the **effectiveness** of the IHSS intervention
3. *The community perspective* we will use the impact of substance use related health system usage in the year following treatment completion as an indication of the **impact** of the intervention on health system outcomes.

Specific variables to address patient wellbeing will be captured in the patient satisfaction survey and during patient aftercare follow up. Anonymized linked data outcomes from ICES will include primary care visits, mental health visits, initiation of opioid agonist therapy, emergency department visits, hospitalizations, and all-cause mortality.

A qualitative thematic analysis will be performed using the text of each pre-group individual interview, the Sharing Circles, and the facilitator interviews. Thematic analysis is a search for themes that emerge as important to the description of the phenomenon [50]. A thematic analysis will allow for a broad exploration and analysis of the impact of the Seeking Safety Sharing Circles and traditional healing methods. The process will be extensive and will include an interpretative analysis of the underlying meanings. This will be further analyzed by means of the iterative process of understanding [50–52].

Interpretation of the text will be a dialectic undertaking, shifting from understanding to explanation, and from explanation to comprehension. The process will involve the identification of themes through “careful reading and re-reading of the data” [53]. There will be a form of pattern recognition within the data, where emerging themes could become the categories for analysis. A coding process will be employed to categorize the transcribed data to reveal common themes. The interpretation process will begin with reading the text several times to gain an understanding of the entire questionnaire responses.

### Propensity score matching

Logistic regression will be used to define propensity scores for treatment groups (pre and post intervention). The treatment group will be matched 1:1 to control using a caliper width of 0.20 greedy matching [54]. Standardized differences (d) will be calculated for each covariate before and after matching. Standardized difference (d) less than 0.10 will indicate balanced covariates [55]. Propensity score matched data will be analyzed to calculate the effect of receiving treatment post-intervention on each helth system usage outcome using McNemar’s test [56]. Proportions will be used to calculate clinical descriptors including relative risk (RR) and number needed to treat or number needed to harm (NNT/NNH), along with 95% confidence intervals (CI) for each descriptor calculated. All statistical analysis will be conducted on the remote server using SAS Version 9.4 [57]. Data will be reviewed by ICES to insure privacy standards are met.

## Discussion

There is a lack of sufficient evidence from quantitative and qualitative research studies about the needs and outcomes of cultural interventions, cost-effectiveness of multi-component treatments, and approaches to the delivery of Indigenous residential treatment programs in Canada. Furthermore, the anecdotal claims and divergent views regarding the effectiveness and appropriateness of Western and Indigenous and innovative methods of treatment approaches needs to be addressed. Other factors includes the poorly defined and lack of specific, evidence-based features of Indigenous residential programs [15, 33, 58–60]. This study will seek to increase the quantity and methodological quality of evaluations of Indigenous residential rehabilitation services at Benbowopka by developing a collaborative partnership between the North Shore Tribal Council and Benbowopka. With this initial study and working together in partnership, the hope we will be develop models of care that synthesise the views of clients and service providers with existing research evidence, including both descriptive data and evaluations of treatment outcomes [61]. Furthermore, findings from this study could add to the knowledge of the need for the utilization of a Two-Eyed Seeing approach and harm reduction in Indigenous residential treatment programs in northern Ontario. Also, this study could identify the growing need for consensus-based best practice treatment guidelines particularly in residential addiction treatment programs [9–11, 62]. As wellness, training, and capacity building will be the cornerstones of this research, we anticipate that the results from this study will help service providers, community organizers and health policymakers make informed decisions on how to improve the current provision of residential addiction treatment programs, particularly those programs that provide both Indigenous healing practices and harm reduction approaches. This evidence could inform future decisions and evidence for treatment approaches for both IGT and SUD across Northern Ontario communities [13, 14, 24, 46].

For Benbowopka, participation in this research will enable enhanced ease of program evaluation, reporting, and future quality improvement and research initiatives. Outcomes of the proposed collaboration will use a *push, pull, and exchange* strategy to benefit the Mamaweswen, Benbowopka, and stakeholders who have an interest in programming and implementation of IGT and SUD treatment. Collaboratively, we will actively disseminate the study findings to stakeholders, including Indigenous health authorities, provincial and federal funding agencies, and community-based treatment programs. Specifically, we will organize in-person presentations to the Chiefs of Ontario and Mamaweswen North Shore Tribal Council. Data will be provided to stakeholders via print, digital, and social media (Facebook/Twitter) via presentation, infographic and explainer video format.

There are a few limitations to this study. Evidence-based Indigenous residential treatment programs for adults with SUD in northern Ontario will lead to improved community benefit. The IHSS treatment model utilizes a culturally appropriate and culturally informed collaborative approach; as such, we should attempt to reach rural and remote northern Ontario communities, including the Treaty 9 First Nation reserves. We also need an understanding of the needs of young adults with IGT and SUD for future treatment programs conducted in northern Ontario with a focus on addressing the lack of evidence of treatments offered.

## Conclusion

This research could highlight the ways in which Indigenous knowledge and Western knowledge could co-exist in an Indigenous residential addiction treatment program. Furthermore, the goal of this research will be to engage and encourage an Indigenous residential treatment program into improved wellness, training, and capacity building with the community leading this research. This evidence could inform future decisions and evidence for treatment approaches for both IGT and SUD across northern Ontario communities. The research methodologies could help to demonstrate how Two-Eyed Seeing Indigenous decolonizing research could occur. Our hope is that this research will further inspire trauma and substance use residential treatment facilities (and society) to become more compassionate and understanding toward this population and Indigenous peoples across Canada, especially those in remote and rural areas.

## Abbreviations

IGT: Intergenerational trauma
SUD: Substance use disorders
IH: Indigenous Healing
NNADAP: National Native Alcohol and Drug Abuse Programs
SS: Seeking Safety
IHSS: Indigenous Healing and Seeking Safety
OHIP: Ontario Health Insurance Plan
ICES: Institute for Clinical Evaluative Sciences
PTSD: Post-Traumatic Stress Disorder
DUSI: Drug Use Screening Inventory
DUSI-R: Drug Use Screening Inventory revised version
CAMH: Center for Addiction and Mental Health
NACRS: National Ambulatory Care Reporting System
DAD: Discharge Abstract Database
RPDB: Registered Persons Database
RR: Relative risk
NNT/NNH: Number needed to treat or number needed to harm
CI: Confidence intervals
OAT: Opiate Agonist Treatment
